# Supplementary figures and images for: COP5/HKR1 changes ciliary beat pattern and biases cell steering during chemotaxis in Chlamydomonas reinhardtii
Source: Sci Rep. 2024 Dec 5;14:30354. doi: 10.1038/s41598-024-81455-2 (PMC11621555; doi:10.1038/s41598-024-81455-2)

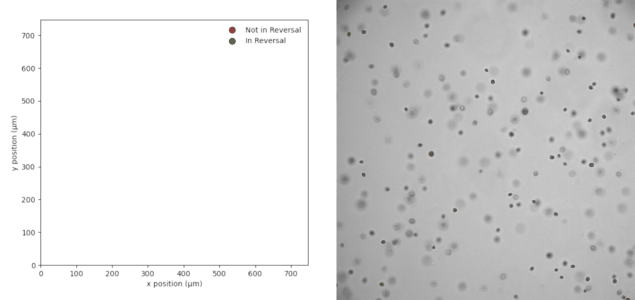

Supplement: Supplementary file 3 — Supplementary Material 3 [file 41598_2024_81455_MOESM3_ESM.gif]
